# Effects of optic nerve head-related parameters on retinal vessel calibers measurement results on fundus photographs

**DOI:** 10.1186/s12886-022-02428-5

**Published:** 2022-05-12

**Authors:** Aiko Iwase, Tae Tsutsumi, Ryo Kawasaki, Jun Suehiro, Akihiko Sekine, Makoto Araie

**Affiliations:** 1 Tajimi Iwase Eye Clinic, 3-101-1, Hon-machi, Tajimi, Gifu Prefecture, Tajimi, 507-0033 Japan; 2 Tsutsumi Eye Clinic, Nerima-ku, Tokyo, Japan; 3 Department of Informatics, Osaka University Graduate School of Medicine, Suita, Osaka Japan; 4 Topcon Corporation, Itabashi-ku, Tokyo, Japan; 5 Kanto Central Hospital of the Mutual Aid Association of Public School Teachers, Setagaya-ku, Tokyo, Japan

**Keywords:** Central Retinal Artery Equivalent (CRAE), Central retinal vein equivalent (CRVE), Optic Nerve Head-Related Structures, Fundus Photographs

## Abstract

**Background:**

Although relationship between the retinal vessel caliber (RVC) and glaucoma is well known, there has been a paucity of information on the effects of glaucoma-related optic nerve head (ONH) structural factors on the RVC. Information on this relationship should be useful in studying possible roles of ocular circulation in the development and progression of glaucoma.

**Method:**

Subjects were from Kumejima Study participants aged 40 years and older in Kumejima, Japan. Normal subjects and eyes were defined according to standardized systemic and ocular examinations. The central retinal artery and vein equivalents (CRAE and CRVE) were determined on fundus photographs by correcting the magnification using the refractive error, corneal curvature, and axial length (AL) of an individual eye and paraxial ray tracing; the ONH structural parameters were determined by planimetry.

**Results:**

In a total of 558 right eyes (558 normal subjects), aged 49.0 ± 7.1 (standard deviation) years with gradable photographs and planimetric results, CRAE averaged 136.1 ± 12.3 μm and CRVE 216.9 ± 17.4 μm. After adjustment for the effects of confounding factors in multivariate analysis, the AL (*P* < 0.001), rim area (*P* = 0.019), disc area (*P* = 0.042), and smoking (*P* = 0.035–0.043) correlated positively, and the mean blood pressure (*P* < 0.001) negatively with CRAE; the AL (*P* < 0.001), rim area (*P* = 0.001), disc area (*P* = 0.005), smoking (*P* < 0.001), and male sex (*P* = 0.013) correlated positively, and the β-peripapillary atrophy (β-PPA) area (*P* = 0.044), vertical Cup/Disc ratio (v-C/D) (*P* = 0.035), and age (*P* < 0.001) negatively with CRVE.

**Conclusion:**

The current study showed significant effects of rim area, v-C/D or β-PPA area determined on the photographs on the RVC measurement results. Further, it showed a necessity to incorporate the glaucoma-related ONH structural parameters as co-variables to correctly estimate the effects of various factors on the RVC.

**Supplementary Information:**

The online version contains supplementary material available at 10.1186/s12886-022-02428-5.

## Background

Retinal vessel caliber (RVC) is a simple and clinical useful index reflexing cardiovascular disorders and a variety of demographic and life-style factors and systemic medical conditions [1–3]. The RVC also is affected by a variety of ocular disorders [4, 5]. Glaucoma, a leading cause of blindness worldwide [6], affects more than 100 million people [7]. It is interesting that cross-sectional population-based studies have agreed on the association of decreased RVCs and glaucoma [8–10], and a prospective population-based study showed that decreased RVCs at baseline were associated with incident primary open-angle glaucoma [11].

The classic hallmarks of glaucoma, which are still relied on during screening in the community, are a reduced optic nerve head (ONH) rim area and increased vertical cup/disc ratio (v-C/D) [12]. Since evidence has suggested an association between vascular risk factors and development and progression of glaucoma [13], and RVCs reflect various circulatory parameters in vivo [1–3], an association between the RVC changes and the ONH rim area or v-C/D, if it exists, would be of clinical interest, not only to study relationship between glaucoma and vascular factors, but also to upgrade estimation of the effects of various systemic factors on the RVC measurement results [1–3] by correcting it effects. Previous studies have reported an association between the RVC changes and the rim area, cup area, v-C/D, and optical coherence tomography (OCT)-measured average retinal nerve fiber layer thickness (RNFLT) in children without glaucoma [14–16] and with the average RNFLT measured using the Heidelberg retina tomography (HRT) [17] or OCT [10] in adult subjects without glaucoma. However, an association between the RVC changes and the rim area, v-C/D, or other ocular parameters related to glaucoma, such as β-peripapillary atrophy (β-PPA) [18–20] that could be measured on the same photographs has not been studied in an adult population that is more likely to undergo screening for systemic and ocular disorders.

For accurate measurements of the RVCs in individual eyes, it is essential to correct the magnification of the image captured by the fundus camera on an individual basis and meet the following two requirements: the magnification must be corrected individually when the proper focus is achieved using the entire subject eye-fundus camera optical system and the RVCs must always be measured in an area at the same distance (absolute distance) from a fundus benchmark. We recently developed a new semiautomatic method that satisfies these two requirements in which the magnification corrections in all subjects’ eyes were individually done when the focusing condition in the combined subject eye-fundus camera optical system was fulfilled and the annular measurement zone was located at the same predetermined absolute distance from the disc center [21]. The purpose of the current study is to study the effects of the v-C/D, rim area, β-PPA area, and other ocular factors on the RVC measured on the same photographs in otherwise normal eyes of adult Japanese subjects after adjustment for other confounding effects.

## Methods

### Study population

The Kumejima Study was a population-based cross-sectional eye disease survey targeting all residents aged 40 years or older in Kumejima Island in Okinawa (Ryukyu) Prefecture, Japan [22, 23]. The study adhered to the tenets of the Declaration of Helsinki and the municipal law of Kumejima Town for protecting private information, and the study protocol was approved by the ethics board of the regional council and the ethics committee of Kumejima Town. Written informed consent was provided by the all participants before being enrolled in the study.

### Study subjects

Experienced ophthalmologists and examiners conducted the screening examinations, which consisted of a structured interview; and measurements of body weight, height, and systemic blood pressure; and ocular examinations. The ophthalmic examinations included measurements of the uncorrected and best-corrected visual acuity (VA), refraction, intraocular pressure (IOP) using a Goldmann applanation tonometer, central corneal thickness (CCT) by specular microscopy (SP-2000, Topcon), and anterior chamber depth (ACD) and axial length (AL) using the IOLMaster (Carl Zeiss Meditec, Dublin, CA), and slit-lamp examination, gonioscopy, ophthalmoscopy, fundus photography, and visual field (VF) testing. One experienced technician obtained a pair of sequential stereoscopic ONH photographs at a parallax of about 8 degrees (30-degree angle of view) and non-stereoscopic fundus photographs (45-degree angle of view) using a digital non-mydriatic fundus camera (TRC-NW7, Topcon, Tokyo, Japan) in both eyes of the subjects. The VF was examined using frequency doubling technology perimetry with the C-20–1 test program (Carl Zeiss Meditec).

Participants were referred for a definitive examination if they were suspected of having ocular abnormalities including glaucoma after meeting one or more of the following criteria: a corrected VA < 20/30, IOP > 19 mmHg, v-C/D ≥ 0.6, superior or inferior (11–1 or 5–7 o’clock hours) rim width/disc diameter ≤ 0.2, bilateral asymmetry of the v-C/D ≥ 0.2, a nerve fiber layer defect or disc hemorrhage, abnormal findings on the slit-lamp examination or fundus photographs, angle width of grade ≤ 2 (van Herick method), and at least one abnormal test point (*P* < 0.05) in the C-20–1 test results. The definitive examination included detailed slit-lamp, gonioscopy, and fundus examinations and VF testing with the Humphrey Field Analyzer 24–2 Swedish Interactive Thresholding Algorithm Standard program (Carl Zeiss Meditec). The diagnoses of glaucoma were based on the clinical records obtained during all examinations and the International Society of Geographic and Epidemiologic Ophthalmology Criteria [12].

### Measurement of RVC

The details of the current method of RVC measurement were described previously [21]. The optical properties of the digital non-mydriatic fundus camera (TRC-NW7) used are available from the manufacturer. An approximate optical model of each subject’s eye was constructed and the refractive error, corneal curvature, and AL were applied to a Gullstrand’s schematic eye [24], and the positions of the focusing lenses in the fundus camera optical system were adjusted according to the refractive error of each subject’s eye. The magnification correction factor was calculated based on this final condition [21]. Eyes with an intraocular lens were excluded because the parametric values of the Gullstrand’s schematic eye for such eyes have not been established.

A binary image was obtained from the fundus photograph (supplemental Figs. 1A and 1B) and an island-like area was defined as a potential optic disc location. Next, its margin automatically was determined by 8 equidistant points located on the outline of the potential and spline curve. This potential disc margin displayed on a computer display was superimposed on the original fundus photograph and an examiner manually corrected it, if needed, by moving the 8 points. After applying the optical magnification correction determined as above, the analysis zone was set as an annular area with an absolute diameter from 1.8 to 2.7 mm (1 to 1.5 times the average Japanese disc diameter [25]), centered on the geometrical disc center automatically determined as the benchmark. Thus, the analysis zone should be at a constant distance from the bench mark, that is, the geometrical disc center, regardless of the disc size or optical magnification of the photograph, and superimposed also on the original fundus photograph (supplemental Fig. 1C). A binary image where blood vessels are white was constructed by means of the binarizing process, which recognized the second derivative of the edge of a vessel. By means of the thinning processing, blood vessels having a length shorter than 10 pixels (corresponding to < 0.1 mm) were eliminated [26] (supplemental Fig. 1D). Further, those with branching parts detected within the analysis zone were eliminated and excluded from further analysis [26]. The width of the vessel area was measured perpendicular to the direction of the vessel, and average width of the vessel was calculated by averaging widths measured along the vessel within the analysis zone. All of the vessels within the analysis zone were measured, and those with magnification-corrected calibers smaller than 60 μm excluded. Arterioles and venules were identified by defining characteristics such as existence and intensity of blood column reflection, hue and clarity, and CRAE and CRVE were calculated using the six biggest arterioles and venules according to the revised formula of Knudtson et al. [21] (supplemental Fig. 1E). Details of this method (Automated Vasculature Analysis by Topcon, AVANT) was reported previously [21].

The intra-examiner and inter-examiner intra-class correlation coefficients for the CRAE and CRVE measurements of the current study were greater than 0.978 [21]. The new method yielded CRAEs and CRVEs that agreed well with the Interactive Vessel Analysis [26] in eyes with ALs of about 24 mm, while the current method yielded smaller values in eyes with shorter ALs and vice versa [21]. This new method should yield more accurate measurement results for Asian populations in which the prevalence of myopia or eyes with long ALs is high [27].

### Planimetry of fundus photographs

The details of the current planimetric method and the reproducibilities of its measurement results have been reported elsewhere [28, 29]. All stereo photographs obtained when the plain fundus photographs used for RVC measurements were taken were re-evaluated by an experienced investigator (T.T.). While stereoscopically viewing the photograph, the inner boundary of the peripapillary scleral ring (clinical disc margin), the cup contour was determined as previously reported [28, 29] and the disc center was determined as the geometrical center of the disc area. The β-PPA area characterized by visible sclera and large choroidal vessels owing to the absence of retinal pigment epithelium was also determined as a closed curve by placing a number of points placed on the outer boundary of the β-PPA area and that of the clinical disc margin. After correcting for magnification by the corneal curvature, AL, and refractive error, the disc center-fovea distance, disc area, rim area, cup area, v-C/D, and β-PPA area were calculated.

### Data analysis

Multiple regression analysis was adopted where the dependent variable was CRAE or CRVE and explanatory variables were age, sex, body mass index (BMI), mean blood pressure, presence or absence of diabetes mellitus, family history of glaucoma, smoking, AL, IOP, disc center-fovea distance, disc area, rim area, cup area, v-C/D, and β-PPA area. JMP® Pro 13 (SAS Institute Inc., Cary, NC) was used for the calculations. *P* < 0.05 was considered statistically significant.

## Results

In the Kumejima Study, reliable results of fundus planimetry were obtained from one eye of 280 and both eyes of 2,194 subjects, after excluding eyes with any systemic and/or ocular abnormalities, those with pseudophakia, aphakia, high myopia (spherical equivalent refraction < -8 diopters and/or AL > 26.5 mm) or high hyperopia (spherical equivalent refraction >  + 5D and/or AL < 21.0 mm), and apparently normal fellow eyes of those with glaucoma or suspected glaucoma. Since the right eye was photographed first, the photographs of the right eyes were adopted. In the current study, the fundus photographs with a 45-degree visual angle were centered on the midpoint between the fovea and disc center, and, thus, the image quality of the nasal portion of an annular analysis area was not sufficiently satisfactory in a significant number of eyes. After further excluding these eyes, 558 normal right eyes of 558 normal subjects were used for RVC measurements (Table 1).

In a separate group of 30 eyes of 30 normal Kumejima Study participants, two investigators (A.I. and M.A.) independently evaluated disc, rim, cup areas, v-C/D and β-PPA area using the current planimetric method. Intra-examiner intra-class correlation coefficients were 0.964 (95% confidence interval: 0.912-0.985) for A.I. and 0.984 (0.961-0.994) for M.A., and inter-examiner intra-class correlation coefficient was 0.992 (0.079-0.997), respectively for disc area. Corresponding figures were 0.789 (0.547-0.910), 0.876 (0.717-0.948) and 0.886 (0.786-0.953), respectively, for rim area, 0.816 (0.599-0.912), 0.883 (0.732-0.952) and 0.846 (0.652-0.936), respectively for cup area, and 0.771 (0.421-909), 0.719 (0.423-0.878) and 0.729 (0.432-883), respectively, for v-C/D and 0.946 (0.840-0.983), 0.864 (0.639-954), and 0.883 (0.644-965), respectively for β-PPA area.

**Table 1:** Subject Characteristics

No. right eyes (subjects)	558(558)
Male/female	258/300
Age (years)	49.0 ± 7.1
Height (cm)	158.4 ± 8.4
Body weight (kg)	62.1 ± 11.4
Body mass index	24.7 ± 3.5
Systolic blood pressure (mmHg)	127.4 ± 19.1
Diastolic blood pressure (mmHg)	75.3 ± 11.1
Mean blood pressure (mmHg)	92.7 ± 12.7
Mean ocular perfusion pressure (mmHg)	46.9 ± 8.3
Family history of glaucoma ( +)	63 of 558
Diabetic mellitus ( +)	21 of 558
Smoking ( +)	230 of 558
Central cornea thickness (μm)	515 ± 33
Intraocular pressure (mmHg)	14.9 ± 2.6
Axial length (mm)	23.6 ± 0.8
Spherical equivalent refraction (diopters)	-0.45 ± 1.44
Optic disc size (mm^2^)	2.53 ± 0.49
Cup area (mm^2^)	0.85 ± 0.38
Neuroretinal rim area (mm^2^)	1.68 ± 0.28
β-peripapillary atrophy (mm^2^)	0.17 (0.00, 0.42)*
Vertical cup/disc ratio	0.54 ± 0.09
Disc center-fovea distance (mm)	4.79 ± 0.29
Central retinal artery equivalent (μm)	136.1 ± 12.3
Central retinal vein equivalent (μm)	216.9 ± 17.4
Arteriolar-to-venular caliber ratio	0.630 ± 0.060

Figures are mean ± standard deviation. *: Median (quartile)

**Table 2:** Effects on central retinal artery equivalent. results of multiple regression analysis including rim area and cup area as explanatory variables

Factors	Partial Regression Coefficient	Standard Error	*P* Value
Age (years)	-0.128*	0.075*	0.0862*
Mean blood pressure (mmHg)	-0.180	0.043	< 0.0001
Smoking ( +)	1.090	0.052	0.0353
Axial length (mm)	3.97	0.64	< 0.0001
Rim area (mm^2^)	4.35	1.85	0.0188
Cup area (mm^2^)	-0.59	1.33	0.6538

Results of multiple regression analysis including smoking, but not sex, as a systemic explanatory variable are shown. Factors with *P* values < 0.010 except for cup area are shown. Results obtained for mean blood pressure, smoking axial length and rim area were essentially the same as when sex, but not smoking, was included as an explanatory variable. *: sex, but not smoking, was included as an explanatory variable. When smoking, but not sex, was included as an explanatory variable, *P* value was 0.1194

**Table 3:** Effects on central retinal artery equivalent. results of multiple regression analysis including disc area and vertical cup-to-disc ratio as explanatory variables

Factors	Partial Regression Coefficient	Standard Error	*P* Value
Age (years)	-0.130*	0.075*	0.0832*
Mean blood pressure (mmHg)	-0.178	0.043	< 0.0001
Smoking ( +)	1.05	0.52	0.0429
Axial length (mm)	3.88	0.63	< 0.0001
Disc area (mm^2^)	2.20	1.20	0.0667
	2.45*	1.20*	0.0421*
v-C/D	-11.3	6.5	0.0822

Results of multiple regression analysis including smoking, but not sex, as a systemic explanatory variable are shown. Factors with *P* values < 0.010 are shown. Results obtained for mean blood pressure, smoking, axial length and v-C/D were essentially the same as when sex, but not smoking, was included as an explanatory variable. *: sex, but not smoking, was included as an explanatory variable. For age, *P* values was 0.1121, when smoking, but not sex, was included as an explanatory variable. V-C/D = vertical cup-to-disc ratio

**Table 4:** effects on central retinal vein equivalent. results of multiple regression analysis including rim area and cup area as explanatory variables

Factors	Partial Regression Coefficient	Standard Error	*P* Value
Age (years)	-0.355	0.097	0.0003
Body mass index	0.38	0.20	0.0567
Smoking ( +)	3.53	0.67	< 0.0001
Axial length (mm)	8.37	0.83	< 0.0001
Rim area (mm^2^)	7.89	2.41	0.0011
β-PPA area (mm^2^)	-3.32	1.76	0.0604
Male gender	1.83*	0.71*	0.0106*
Cup area (mm^2^)	-0.10	1.73	0.9525

Results of multiple regression analysis including smoking, but not sex, as a systemic explanatory variable are shown. Factors with *P* values < 0.010 except for cup area are shown. Results obtained for the factors except for sex were essentially the same as when sex, but not smoking, was included as an explanatory variable. *: sex, but not smoking, was included as an explanatory variable. β-PPA = β-peripapillary atrophy

**Table 5:** Effects on Central Retinal Vein Equivalent. Results of Multiple Regression Analysis Including Disc Area and Vertical Cup-to-Disc Ratio as Explanatory Variables

Factors	Partial Regression Coefficient	Standard Error	*P* Value
Age (years)	-0.359	0.097	0.0002
Smoking ( +)	3.47	0.68	< 0.0001
Axial length (mm)	8.23	0.83	< 0.0001
Disc area (mm^2^)	4.40	1.56	0.0050
v-C/D	-17.84	4.47	0.0349
β-PPA area (mm^2^)	-3.58	1.77	0.0437
Male sex	1.78*	0.71*	0.0128*

Results of multiple regression analysis including smoking, but not sex, as a systemic explanatory variable are shown. Factors with *P* values < 0.010 are shown. Results obtained for the factors except for sex were essentially the same as when sex, but not smoking, was included as an explanatory variable. *: sex, but not smoking, was included as an explanatory variable. For age, v-C/D = vertical cup-to-disc ratio; β-PPA = β-peripapillary

The average CRAE was 136.1 ± 12.3 (standard deviation, *n* = 558) microns, the average CRVE was 216.9 ± 17.4 microns, and the arteriolar-to-venular caliber ratio was 0.630 ± 0.060. The results of simple regression analysis treating CRAE or CRVE as a response parameter and factors listed in Table 1 as an explanatory variable are shown in Supplemental Tables 1A and 1B. Among the explanatory variables, high inter-correlations were seen between the disc area and rim area, between the disc area and cup area, between the cup area and v-C/D and between sex and smoking (Pearson’s correlation coefficient, 0.635, 825 and 0.817 and polychoric correlation coefficient, 0.729). Therefore, the effects of the rim area and cup area, those of the disc area and v-C/D, that of sex and that of smoking were separately included in the analysis.

After adjusting for the confounding effects of other factors, the AL (*P* < 0.001), rim area (*P* = 0.020) and disc area (*P* = 0.042 when sex, but not smoking, was included as an explanatory variable) significantly positively correlated with the CRAE. Among the systemic factors, mean blood pressure (*P* < 0.001) significantly negatively and smoking significantly positively (*P* = 0.0353–0.0429) correlated with the CRAE (Tables 2, 3).

The AL (*P* < 0.001), rim area (*P* = 0.001), and disc area (*P* = 0.005) and the β-PPA area (*P* = 0.044 when sex, but not smoking, was included as an explanatory variable) significantly positively, and v-C/D (*P* = 0.0349) significantly negatively correlated with the CRVE. Among the systemic factors, age (*P* < 0.001) significantly negatively and smoking (*P* < 0.001) significantly positively correlated with the CRVE. The male sex (*P* = 0.0110–0.0128) significantly positively correlated with marginal significance with the CRVE Tables 4, 5.

## Discussion

The RVC reflects the vascular structural conditions depending on multiple systemic and local pathophysiologic factors [1–3]. Those affecting the retinal arteriolar caliber, i.e., CRAE, or the retinal venular caliber, i.e., CRVE, are not the same [1]. In the current normal eyes of Japanese adults (mean age, 49 years), decreased disc and rim area and greater v-C/D were associated significantly with decreased CRAE and CRVE measurement results, and greater β-PPA area with decreased CRVE measurement results, after adjusting for other confounding factors’ effects. The correlation between age, blood pressure, smoking, BMI, sex or disc area with CRAE or CRVE measurement results currently found agreed with many previous studies performed in adult subjects or children [1, 2, 14, 16, 30].

Reduced CRAE was associated with incident POAG in a 10-year prospective follow-up [11], while reduced rim area and greater v-C/D were associated with reduced CRAE in the current normal adults. Thus, it may be tempting to assume that the current normal subjects with a relatively smaller rim area and greater v-C/D with reduced CRAE or CRVE might be associated with early vascular dysfunction [13] and that they are relatively more vulnerable to glaucomatous insults; such eyes encountered during community screening need closer future monitoring. This current finding is comparable to the significant positive correlation between CRAE or CRVE and OCT- or HRT-measured average RNFL thickness in eyes of subjects without glaucoma with a mean age of 54 to 57 years [17, 31]. However, a positive correlation between the CRAE or CRVE and rim area or average RNFL thickness and a negative correlation between the CRVE and v-C/D, also were seen in children without ocular and systemic diseases [14–16], which may suggest that the correlation of the RVCs with rim area or v-C/D currently found in normal adults may be partly explained by anatomic and physiologic relationships between nutritional support and relevant tissue area, or that a reduced rim area or greater v-C/D indicates a less crowded disc causing less vascular compression at the level of the ONH.

A large β-PPA area is associated with greater glaucomatous damage and progression [18–20], and β-PPA may be partly related to an IOP-independent damaging mechanism of POAG in Japanese [32]. The current study reported possible association of a larger β-PPA area and decreased CRVE in apparently normal adults after adjustment for other confounding factors’ effects. Distinct genetic factors are associated with the CRAE and CRVE [33] and there is less understanding of the CRVE pathophysiology [1]. Previous studies have reported that an association between systemic inflammation or obesity was evident with CRVE more than with CRAE [1]. Although complete exclusion of the speculation that β-PPA and decreased CRVE reflected subclinical circulatory dysfunction is difficult, there may be another possibility. Since retinal veins are thought to be mechanically less resistant than retinal arteries (Roggendorf & Cervos-Navarro. 1977), vascular compression at the level of the ONH, if present, would affect the CRVE more than the CRAE as suggested by a negative correlation between v-C/D and CRVE in healthy children [15]. A larger β-PPA may be related to structural changes at the ONH and less compression at that level. This effect on the CRVE, if it existed, however, is thought be corrected by a significant negative correlation between the CRVE and v-C/D found in the current analysis.

In the current subjects, a longer AL was associated with increased CRAE and CRVE. It may be difficult to speculate about the underlying mechanisms of this result, which did not agree with the result obtained previously [34]. The AL should have the strongest effect on the magnification of the fundus image [24], and the current method should have improved the correction of the effect of AL on the magnification by the original Littmann’s method [24] by incorporating the AL of the individual eyes and adopting the ray-tracing method [21]. It is possible to re-calculate the RVCs of the current subject eyes without using individually measured AL but rather by assuming the mean AL of the study population of 23.6 mm (Table 1) as in the previous method [26]. The calculated CRAEs and CRVEs were negatively correlated with the AL (≈-3.0 mμ/mm of AL), which agreed with the previous study [34]. There is a possibility that the current method [21] over-corrected the effect of AL on the magnification. The optical system of Topcon fundus camera currently used is not exactly same to that of Zeiss fundus camera. Based on detailed information on the optical system of the Topcon fundus camera currently used [21] and AL, refractive error and corneal curvature of each subject eye, it is possible to calculate the magnification correction factor using the original Littmann method [24] (Supplemental tables 2A-2D). The obtained results using magnification correction of Littmann method were similar to those obtained using the method currently used [21], and measured CRAE and CRVE still showed significant positive correlation with AL (Supplemental tables 2A-2D). Thus, it seems unlikely that a significant positive correlation between AL and measured CRAE and CRVE currently obtained reflected an artefact in the method of magnification correction currently used. Recently, Wen et al. [35] studied the effects of AL on the superficial vessel density determined using optical coherence tomographs angiography using magnification correction of Littmann method [24] based on AL, refractive error and corneal curvature of a subject eye. They found that the vessel density was lower with a longer AL in the parafoveal region, which was suggested to be attributable to stretching of inner retina associated with a longer AL [35]. If the inner retina is stretched, a retinal arteriole or venule may be also stretched and its measured calibers be changed without significantly affecting its cross-sectional area. Since CRAE or CRVE is calculated based on the measured calibers of 6 representative retinal arterioles or venules, respectively, a larger CRAE or CRVE measurement results with a longer AL may not necessarily indicate increased cross-sectional area of CRAE or CRVE with a longer AL. Rather, the positive correlation of the measured CRAE and CRVE with AL currently found is thought to indicate the necessity to correct this apparent effect of AL in analyzing the effects of various systemic or ocular factors on the optically determined CRAE and CRVE.

The current study had limitations. The current subjects represented only 27% of ophthalmologically normal subjects of the Kumejima study for which reliable fundus photographs used to determine CRAE and CRVE were obtained, and, thus, the current results may not be directly applicable to the general population of Kumejima. If we compare the current subjects to normal participants of the Kumejima Study [29], most of the parametric values were very similar, but the mean age was younger (48.9 vs. 56.8 years), the mean blood pressure lower (93.9 vs. 98.9 mmHg), and the β-PPA area smaller (0.30 vs. 0.47 mm^2^) in the current subjects. This small difference is thought to be corrected by incorporating these parameters as explanatory variables. Although the mean values of CRAE and CRVE of the current subjects may not be exactly the same to those of the whole normal participants of the Kumejima Study, as far as the results obtained for the effects of various ocular factors on the CRAE and CRVE were concerned, the obtained results were thought to be less affected by selection bias. The current β-PPAs were determined photographically and, thus, probably included a γ-zone free of Bruch’s membrane [36]. The characteristics of the β-PPAs might have been evaluated better by spectral-domain OCT, which was unavailable at the time when the Kumejima Study was designed. However, in routine clinical practice, the correlation between the RVCs and the β-PPA size determined photographically, which is still widely used, would be of practical and clinical use for physicians. It seems rather exceptional to adopt OCT-based β-PPA in routine clinical practice.

The RVC measurement is a simple non-invasive method to assess circulatory status in the ocular fundus in vivo and has been used widely to assess systemic, environmental, genetic, and ocular risk factors. The current study confirmed that the RVCs measured on fundus photographs were affected by previously reported factors such as age, blood pressure or smoking and disc area also in the Kumejima Study participants. The significant contribution of the rim area, v-C/D, and β-PPA which are readily available from fundus photographs used to determine the RVCs on the CRAE and/or CRVE measurement results in normal adult eyes was first documented in the current study. Further, the current study first suggested that importance of incorporating AL of an individual eye in correctly estimating the effects of any of the target systemic or ocular factors on the CRAE or CRVE measured on fundus photographs or to compare CRAE or CRVE measurement results inter-individually.

## Conclusions

The current study found that in addition to previously reported systemic factors such as smoking or blood pressure, rim area, v-C/D or β-PPA area showed significant effects on the RVC measurement results. Further, it was thought that these glaucoma-related ONH structural parameters and axial length were needed to be included as co-variables to correctly estimate the effects of various systemic and ocular factors on the RVC.

## Supplementary Information

**Additional file 1:** **Supplemental Tables 1A** Effects on Central Retinal Artery Equivalent. Results of Simple Regression Analysis. Supplemental Table 1B. Effects on Central Retinal Vein Equivalent. Results of Simple Regression Analysis. **Supplemental Table 2A** Effects on Central Retinal Artery Equivalent. Results of Multiple Regression Analysis Including Rim Area and Cup Area as Explanatory Variables and Using Magnification Correction Method According to Littmann (1982). **Supplemental Table 2B** Effects on Central Retinal Artery Equivalent. Results of Multiple Regression Analysis Including Disc Area and Vertical Cup-to-Disc Ratio as Explanatory Variables and Using Magnification Correction Method According to Littmann (1982). **Supplemental Table 2C** Effects on Central Retinal Vein Equivalent. Results of Multiple Regression Analysis Including Rim Area and Cup Area as Explanatory Variables and Using Magnification Correction Method According to Littmann (1982). **Supplemental Table 2D** Effects on Central Retinal Vein Equivalent. Results of Multiple Regression Analysis Including Disc Area and Vertical Cup-to-Disc Ratio as Explanatory Variables and Using Magnification Correction Method According to Littmann (1982).

**Additional file 2:** **Supplemental Figure 1A** Original fundus photograph. **Supplemental Figure 1B** A binary images was created from the original color fundus photograph and potential optic disc location defined as an island-like area. **Supplemental Figure 1C** Thus determined potential disc margin was displayed on a computer display being superimposed on the original fundus photograph. The geometrical center of the disc was set as the benchmark, and after applying the optical magnification correction, the analysis zone was set as an annular area with an absolute diameter from 1.8 to 2.7 mm centered on the disc geometrical center. The analysis zone was at a constant distance from the disc center regardless of the disc size or optical characteristics of the eye, and superimposed also on the fundus photograph. **Supplemental Figure 1D** A binary image where blood vessels are white was constructed by means of the binarizing process, which recognized the second derivative of the edge of a vessel. Blood vessels having a length shorter than 10 pixels (corresponding to < 0.1 mm) were eliminated [26]. In this image, blood vessels with magnification-corrected calibers smaller than 60 μm were also eliminated. **Supplemental Figure 1E** The 6 biggest arterioles and venules are shown, from which central artery and vein equivalents were calculated [21].

## Data Availability

The datasets generated and/or analysed during the current study are not publicly available. The data are from the Kumejima Study and are owned by Japan Glaucoma Society (JGS). Based on the agreement between the ethical committee of Kumejima Town and JGS concluded in May, 2005 and the municipal law of Kumejima Town for protecting private information, access to the original data is restricted to researchers who are members of JGS and are accepted by the society. To manage the epidemiological data which JGS gathered, JGS has the Epidemiology Study Group Data Center, which can be contacted at: epid.jgs@nifty.com. Upon reasonable and official request to the Data Center, anonymized data will be shared with the requester.

## Abbreviations

ONH: Optic nerve head
CRAE: Central retinal artery equivalent
CRVE: Central retinal vein equivalent
v-C/D: Vertical cup/disc ratio
β-PPA: β-Peripapillary atrophy
RVC: Retinal vessel caliber
RNFLT: Retinal nerve fiber layer thickness
POAG: Primary open-angle glaucoma
HRT: Heidelberg retina tomography
OCT: Optical coherence tomography
VA: Visual acuity
CCT: Central corneal thickness
IOP: Intraocular pressure
ACD: Anterior chamber depth
AL: Axial length
VF: Visual field

## References

[1] Sun C, Wang JJ, Mackey DA, Wong TY (2009). Retinal vascular caliber: Systemic, environmental, and genetic associations. Surv Ophthalmol.

[2] Myers CR, Klein R, Knudtson MD, Lee KE, Gangnon R, Wong TY, Klein BE (2012). Determinants of retinal venular diameter: the Beaver Dam Eye Study. Ophthalmology.

[3] Ikram MK, Cheung CY, Lorenzi M, Klein R, Jones TLZ, Wong TY (2013). Retinal vascular caliber as a biomarker for diabetes microvascular complications Diabetes Care.

[4] Jonas JB, Nguyen XN, Naumann GOH (1989). Parapapillary retinal vessel diameter in normal and glaucoma eyes I. Morphometric data Invest Ophthalmol Vis Sci.

[5] Jeganathan VSE, Kawasaki R, Wang JJ (2008). Retinal vascular caliber and age-related macular degeneration: the Singapore Malay Eye Study. Am J Ophthalmol.

[6] Quigley HA, Broman AT (2006). The number of people with glaucoma worldwide in 2010 and 2020. Br J Ophthalmol.

[7] Tham Y-C, Li X, Wong TY, Quigley HA, Aung T, Cheng C-Y (2014). Global prevalence of glaucoma and projections of glaucoma burden through 2040. Ophthalmology.

[8] Mitchell P, Leung H, Wang JJ (2005). Retinal vessel diameter and open-angle glaucoma: the Blue Mountains Eye Study. Ophthalmology.

[9] Amerasinghe N, Aung T, Cheung N (2008). Evidence of retinal vascular narrowing in glaucomatous eyes in an Asian population. Invest Ophthalmol Vis Sci.

[10] Tham Y-C, Siantar RG, Cheung CY-L (2016). Inter-relationships between retinal vascular caliber, retinal nerve fiber layer thickness, and glaucoma: a mediation analysis approach. Invest Ophthalmol Vis Sci.

[11] Kawasaki R, Wang JJ, Rochtchina E, Lee AJ, Wong TY, Mitchell P (2003). Retinal vessel caliber Is associated with the 10-year incidence of glaucoma - The Blue Mountains Eye Study. Ophthalmology.

[12] Foster PJ, Buhrmann R, Quigley HA, Johnson G (2002). The definition and classification of glaucoma in prevalence surveys. Br J Ophthalmol.

[13] Yanagi M, Kawasaki R, Wang JJ, Wong TY, Crowston J, Kiuchi Y (2011). Vascular risk factors in glaucoma: a review. Clin Exp Ophthalmol.

[14] Cheung N, Huynb S, Wang JJ (2008). Relationships of retinal vessel diameters with optic disc, macular and retinal nerve fiber layer parameters in 6-year-old children. Invest Ophthalmol Vis Sci.

[15] Lim LS, Saw SM, Cheung N, Mitchel P, Wong TY (2009). Relationship of retinal vascular caliber with optic disc and macular structure. Am J Ophthalmol.

[16] Samarawickrama C, Hyunb SC, Wang JJ, et al. Relationship between retina structures and retinal vessel caliber in normal adolescents. Invest Ophthalmol Vis Sci. 2009; 50:5619–5624.10.1167/iovs.09-3878 10.1167/iovs.09-3878 19661230

[17] Zheng Y, Cheung N, Aung T, Mitchell P, He M, Wong TY (2009). Relationship Of retinal vascular caliber with retinal nerve fiber layer thickness: The Singapore Malay Eye Study. Invest Ophthalmol Vis Sci.

[18] Park KH, Tomita G, Liou SY, Kitazawa Y (1996). Correlation between peripapillary atrophy and optic nerve damage in normal-tension glaucoma. Ophthalmology.

[19] Teng CC, De Moraes CG, Prata TS, Tello C, Ritch R, Liebmann JM (2010). β-zone parapapillary atrophy and the velocity of glaucoma progression. Ophthalmology.

[20] Kim YW, Lee EJ, Kim TW, Kim M, Kim H (2014). Microstructure of β-zone parapapillary atrophy and rate of retinal nerve fiber layer thinning in primary open-angle glaucoma. Ophthalmology.

[21] Iwase A, Sekine A, Suehiro J (2017). A new method of magnification correction for accurately measuring retinal vessel calibers from fundus photographs. Invest Ophthalmol Vis Sci.

[22] Sawaguchi S, Sakai H, Iwase A (2012). Prevalence of primary angle closure and primary angle-closure glaucoma in a southwestern rural population of Japan: The Kumejima Study. Ophthalmology.

[23] Yamamoto S, Sawaguchi S, Iwase Y (2014). Primary open-angle glaucoma in a population associated with high prevalence of primary angle-closure glaucoma: the Kumejima Study. Ophthalmology.

[24] Littmann H (1982). Zur Bestimmung der wahren Grosse eines Objekts auf dem Hintergrund des lebenden Auges. Klin Monatsbl Augenheilkd.

[25] Tsutsumi T, Tomidokoro A, Araie M, Iwase A, Sakai H, Sawaguchi S (2012). Planimetrically determined vertical cup/disc and rim width/disc diameter ratios and related factors. Invest Ophthalmol Vis Sci.

[26] The Atherosclerosis Risk in Communities (ARIC) Study Research Group. ARIC Retinal Reading Center Protocol, Manual 14B. ARIC Coordinating Center ;1996

[27] Sawada A, Tomidokoro A, Araie M (2008). Refractive errors in an elderly Japanese population: the Tajimi study. Ophthalmology.

[28] Saito H, Tsutsumi T, Iwase A, Tomidokoro A, Araie M (2010). Correlation of disc morphology quantified on stereophotographs to results by Heidelberg Retina Tomograph II, GDx variable corneal compensation, and visual field tests. Ophthalmology.

[29] Iwase A, Sawaguchi S, Sakai H, Tanaka K, Tsutsumi T, Araie M (2017). Optic disc, rim and peripapillary chorioretinal atrophy in normal Japanese eyes: the Kumejima Study. Jpn J Ophthalmol.

[30] Lee KE, Klein BEK, Klein R, Meuer SM (2007). Association of retinal vessel caliber to optic disc and cup diameters. Invest Ophthalmol Vis Sci.

[31] Tham Y-C, Cheng C-Y, Zheng Y, Aung T, Wong TY, Cheung CY (2013). Relationship between vascular geometry with retinal nerve fiber layer and ganglion cell-inner plexiform layer in nonglaucomatous eyes. Invest Ophthalmol Vis Sci.

[32] Mataki N, Tomidokoro A, Iwase A, Araie M (2018). Beta-peripapillary atrophy of the optic disc and its determinants in Japanese eyes: a population-based study. Acta Ophthalmol.

[33] Roggendorf W, Cervos-Navarro J (1977). Ultra-structure of arterioles in the cat brain. Cell Tissue Res.

[34] Lim LS, Cheung CY, Lin X, Mitchell P, Wong TY, Mei-Saw S (2011). Influence of refractive error and axial length on retinal vessel geometric characteristics. Invest Ophthalmol Vis Sci.

[35] Wen C, Pei C, Xu X, Lei J (2019). Influence of axial length and parafoveal and peripapillary metrics from swept source optical coherence tomography angiography. Curr Eye Res.

[36] Jonas JB, Jonas SB, Jonas RA (2012). Parapapillary atrophy: Histological gamma zone and delta zone. PLoS ONE.

