# Supplementary material for: Effects of optic nerve head-related parameters on retinal vessel calibers measurement results on fundus photographs
Source: BMC Ophthalmol. 2022 May 12;22:215. doi: 10.1186/s12886-022-02428-5 (PMC9097128; doi:10.1186/s12886-022-02428-5)
Supplement: Supplementary file 1 — Additional file 1: Supplemental Tables 1A Effects on Central Retinal Artery Equivalent. Results of Simple Regression Analysis. Supplemental Table 1B. Effects on Central Retinal Vein Equivalent. Results of Simple Regression Analysis. Supplemental Table 2A Effects on Central Retinal Artery Equivalent. Results of Multiple Regression Analysis Including Rim Area and Cup Area as Explanatory Variables and Using Magnification Correction Method According to Littmann (1982). Supplemental Table 2B Effects on Central Retinal Artery Equivalent. Results of Multiple Regression Analysis Including Disc Area and Vertical Cup-to-Disc Ratio as Explanatory Variables and Using Magnification Correction Method According to Littmann (1982). Supplemental Table 2C Effects on Central Retinal Vein Equivalent. Results of Multiple Regression Analysis Including Rim Area and Cup Area as Explanatory Variables and Using Magnification Correction Method According to Littmann (1982). Supplemental Table 2D Effects on Central Retinal Vein Equivalent. Results of Multiple Regression Analysis Including Disc Area and Vertical Cup-to-Disc Ratio as Explanatory Variables and Using Magnification Correction Method According to Littmann (1982). [file 12886_2022_2428_MOESM1_ESM.docx]

Supplemental Table 1A. Effects on Central Retinal Artery Equivalent. Results of Simple Regression Analysis

| **Factors** | **Partial Regression Coefficient** | **Standard Error** | ***P* Value** |
| --- | --- | --- | --- |
| Male/female | 0.66 | 0.52 | 0.2072 |
| Age (years) | -0.259 | 0.073 | 0.0006 |
| Height (cm) | 0.168 | 0.62 | 0.0070 |
| Body weight (kg) | 0.041 | 0.046 | 0.3754 |
| Body mass index | -0.11 | 0.15 | 0.4562 |
| Systolic blood pressure (mmHg) | -0.111 | 0.027 | <0.0001 |
| Diastolic blood pressure (mmHg) | -0.110 | 0.047 | 0.0192 |
| Mean blood pressure (mmHg) | -0.141 | 0.041 | 0.0006 |
| Mean ocular perfusion pressure (mmHg) | -0.236 | 0.062 | 0.0001 |
| Family history of glaucoma (+) | 0.79 | 0.82 | 0.3349 |
| Diabetic mellitus (+) | -0.67 | 1.37 | 0.6254 |
| Smoking (+) | 1.20 | 0.52 | 0.0233 |
| Central cornea thickness (μm) | 31.0 | 15.8 | 0.0499 |
| Intraocular pressure (mmHg) | 0.21 | 0.20 | 0.2887 |
| Axial length (mm) | 3.91 | 0.61 | <0.0001 |
| Spherical equivalent refraction (diopters) | -1.20 | 0.36 | 0.0009 |
| Optic disc size (mm^2^) | 0.58 | 1.15 | 0.6170 |
| Cup area (mm^2^) | 0.042 | 1.38 | 0.9759 |
| Neuroretinal rim area (mm^2^) | 2.32 | 1.89 | 0.2197 |
| β-peripapillary atrophy (mm^2^) | 1.14 | 1.34 | 0.3957 |
| Vertical cup/disc ratio | -3.89 | 5.84 | 0.5025 |
| Disc center-fovea distance (mm) | 1.68 | 1.83 | 0.3570 |

Supplemental Table 1B. Effects on Central Retinal Vein Equivalent. Results of Simple Regression Analysis

| **Factors** | **Partial Regression Coefficient** | **Standard Error** | ***P* Value** |
| --- | --- | --- | --- |
| Male/female | 3.71 | 0.73 | <0.0001 |
| Age (years) | -0.57 | 0.10 | <0.0001 |
| Height (cm) | 0.59 | 0.85 | <0.0001 |
| Body weight (kg) | 0.360 | 0.063 | 0.0001 |
| Body mass index | 0.44 | 0.21 | 0.0355 |
| Systolic blood pressure (mmHg) | -0.019 | 0.039 | 0.6330 |
| Diastolic blood pressure (mmHg) | 0.141 | 0.066 | 0.0338 |
| Mean blood pressure (mmHg) | 0.059 | 0.058 | 0.3127 |
| Mean ocular perfusion pressure (mmHg) | 0.080 | 0.089 | 0.3686 |
| Family history of glaucoma (+) | 0.084 | 1.168 | 0.9425 |
| Diabetic mellitus (+) | 1.28 | 1.94 | 0.5094 |
| Smoking (+) | 4.47 | 0.73 | <0.0001 |
| Central cornea thickness (μm) | 24.2 | 22.4 | 0.2793 |
| Intraocular pressure (mmHg) | 0.098 | 0.282 | 0.7277 |
| Axial length (mm) | 8.31 | 0.82 | <0.0001 |
| Spherical equivalent refraction (diopters) | -2.98 | 0.50 | <0.0001 |
| Optic disc size (mm^2^) | 3.08 | 1.63 | 0.0591 |
| Cup area (mm^2^) | 1.51 | 1.96 | 0.4410 |
| Neuroretinal rim area (mm^2^) | 6.19 | 2.66 | 0.0204 |
| β-peripapillary atrophy (mm^2^) | 0.089 | 1.906 | 0.9626 |
| Vertical cup/disc ratio | -5.03 | 8.28 | 0.5438 |
| Disc center-fovea distance (mm) | 2.36 | 2.59 | 0.3617 |

Supplemental table 2A. Effects on Central Retinal Artery Equivalent. Results of Multiple Regression Analysis Including Rim Area and Cup Area as Explanatory Variables and Using Magnification Correction Method According to Littmann (1982)

| **Factors** | **Partial Regression Coefficient** | **Standard Error** | ***P* Value** |
| --- | --- | --- | --- |
| Age (years) | -0.141  -0.153* | 0.075  0.075* | 0.0602  0.0423* |
| Mean blood pressure (mmHg) | -0.182 | 0.043 | <0.0001 |
| Smoking (+) | 1.06 | 0.52 | 0.0418 |
| Axial length (mm) | 2.94 | 0.64 | <0.0001 |
| Rim area (mm^2^) | 4.34 | 1.86 | 0.0184 |
| Cup area (mm^2^) | -0.63 | 1.33 | 0.6356 |

Results of multiple regression analysis including smoking, but not gender, as a systemic explanatory variable are shown. Factors with *P* values < 0.010 except for cup area are shown. Results obtained for mean blood pressure, smoking, axial length and rim area were essentially the same as when gender, but not smoking, was included as an explanatory variable. *: Gender, but not smoking, was included as an explanatory variable.

Supplemental table 2B. Effects on Central Retinal Artery Equivalent. Results of Multiple Regression Analysis Including Disc Area and Vertical Cup-to-Disc Ratio as Explanatory Variables and Using Magnification Correction Method According to Littmann (1982)

| **Factors** | **Partial Regression Coefficient** | **Standard Error** | ***P* Value** |
| --- | --- | --- | --- |
| Age （years） | -0.144  -0.154* | 0.075  0.075* | 0.0559  0.0406* |
| Mean blood pressure (mmHg) | -0.179 | 0.043 | <0.0001 |
| Smoking (+) | 1.02 | 0.52 | 0.0506 |
| Axial length (mm) | 2.86 | 0.64 | <0.0001 |
| Disc area (mm^2^) | 2.21  2.41* | 1.21  1.21 | 0.0679  0.0422* |
| v-C/D | -11.4 | 6.52 | 0.0804 |

Results of multiple regression analysis including smoking, but not gender, as a systemic explanatory variable are shown. Factors with *P* values < 0.010 are shown. Results obtained for mean blood pressure, smoking, axial length and v-C/D were essentially the same as when gender, but not smoking, was included as an explanatory variable. *: Gender, but not smoking, was included as an explanatory variable. V-C/D = vertical cup-to-disc ratio.

Supplemental table 2C. Effects on Central Retinal Vein Equivalent. Results of Multiple Regression Analysis Including Rim Area and Cup Area as Explanatory Variables and Using Magnification Correction Method According to Littmann (1982)

| **Factors** | **Partial Regression Coefficient** | **Standard Error** | ***P* Value** |
| --- | --- | --- | --- |
| Age (years) | -0.396 | 0.098 | <.0001 |
| Body mass index | 0.37 | 0.20 | 0.0599 |
| Smoking (+) | 3.51 | 0.68 | < .0001 |
| Axial length (mm) | 6.75 | 0.84 | < .0001 |
| Rim area (mm^2^) | 7.95 | 2.43 | 0.0011 |
| β-PPA area (mm^2^) | -3.12 | 1.78 | 0.0800 |
| Gender | 1.72* | 0.72* | 0.0172* |
| Cup area (mm^2^) | -0.15 | 1.74 | 0.9338 |

Results of multiple regression analysis including smoking, but not gender, as a systemic explanatory variable are shown. Factors with *P* values < 0.010 except cup area are shown. Results obtained for the factors except for gender were essentially the same as when gender, but not smoking, was included as an explanatory variable. *: Gender, but not smoking, was included as an explanatory variable. β-PPA = β-peripapillary atrophy.Supplemental table 2D. Effects on Central Retinal Vein Equivalent. Results of Multiple Regression Analysis Including Disc Area and Vertical Cup-to-Disc Ratio as Explanatory Variables and Using Magnification Correction Method According to Littmann (1982)

| **Factors** | **Partial Regression Coefficient** | **Standard Error** | ***P* Value** |
| --- | --- | --- | --- |
| Age (years) | -0.400 | 0.098 | < .0001 |
| Body mass index | 0.36 | 0.20 | 0.0690 |
| Smoking (+) | 3.45 | 0.68 | < .0001 |
| Axial length (mm) | 6.60 | 0.83 | < .0001 |
| Disc area (mm^2^) | 4.41 | 1.57 | 0.0053 |
| v-C/D | -17.99 | 8.51 | 0.0349 |
| β-PPA area (mm^2^) | -3.38 | 1.78 | 0.0587 |
| Gender | 1.67* | 0.72* | 0.0207 |

Results of multiple regression analysis including smoking, but not gender, as a systemic explanatory variable are shown. Factors with *P* values < 0.010 are shown. Results obtained for the factors except for gender were essentially the same as when gender, but not smoking, was included as an explanatory variable. *: Gender, but not smoking, was included as an explanatory variable. β-PPA = β-peripapillary atrophy.
