# Supplementary material for: Effects of optic nerve head-related parameters on retinal vessel calibers measurement results on fundus photographs
Source: BMC Ophthalmol. 2022 May 12;22:215. doi: 10.1186/s12886-022-02428-5 (PMC9097128; doi:10.1186/s12886-022-02428-5)

Supplemental Figure 1A


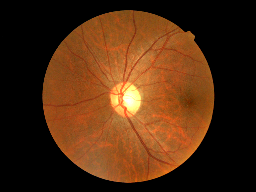


Supplemental Figure 1B


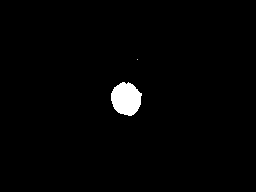


Supplemental Figure 1C


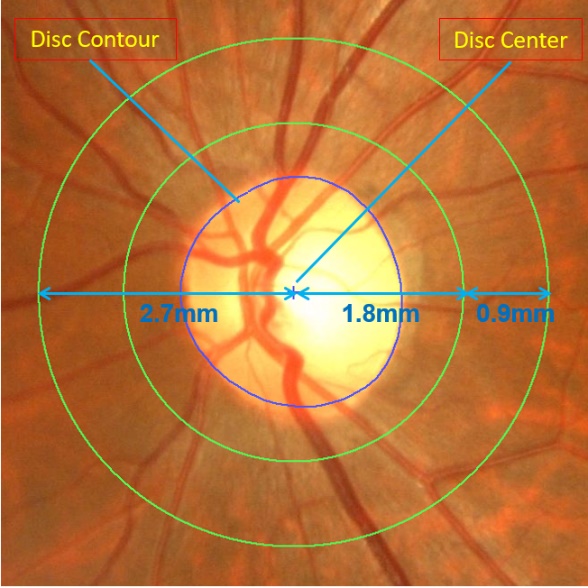


Supplemental Figure 1D


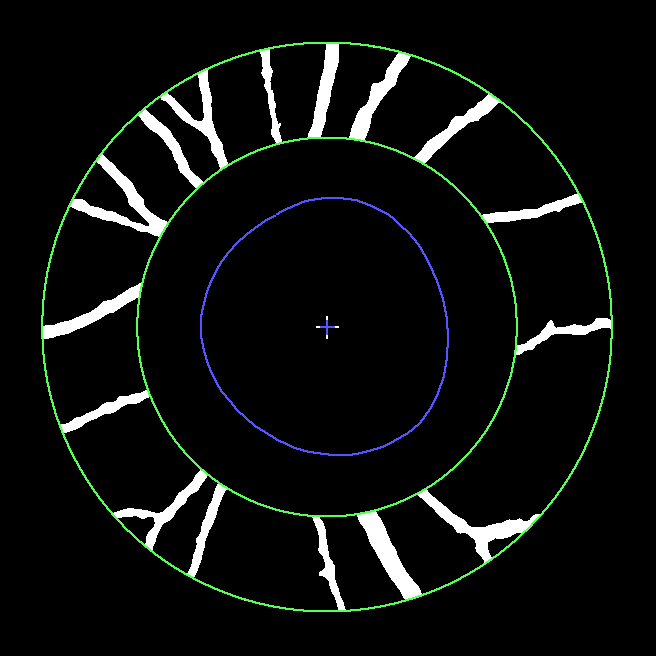


Supplemental Figure 1E


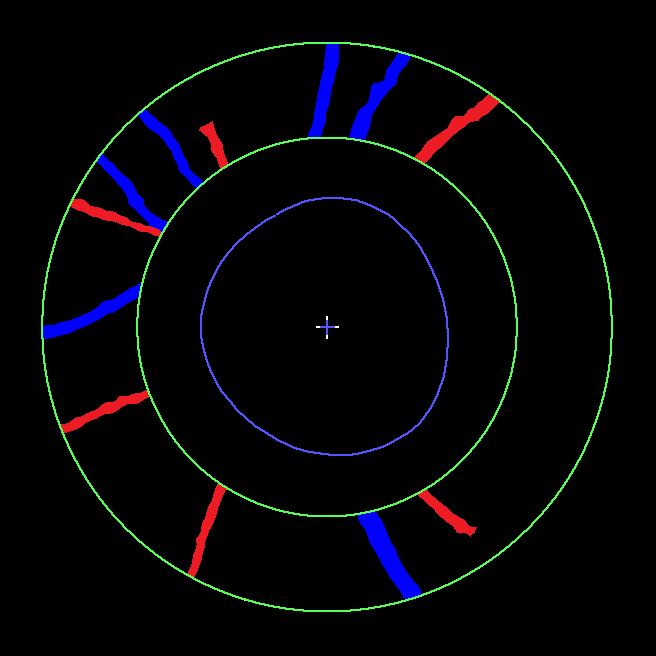

Supplement: Supplementary file 2 — Additional file 2: Supplemental Figure 1A Original fundus photograph. Supplemental Figure 1B A binary images was created from the original color fundus photograph and potential optic disc location defined as an island-like area. Supplemental Figure 1C Thus determined potential disc margin was displayed on a computer display being superimposed on the original fundus photograph. The geometrical center of the disc was set as the benchmark, and after applying the optical magnification correction, the analysis zone was set as an annular area with an absolute diameter from 1.8 to 2.7 mm centered on the disc geometrical center. The analysis zone was at a constant distance from the disc center regardless of the disc size or optical characteristics of the eye, and superimposed also on the fundus photograph. Supplemental Figure 1D A binary image where blood vessels are white was constructed by means of the binarizing process, which recognized the second derivative of the edge of a vessel. Blood vessels having a length shorter than 10 pixels (corresponding to < 0.1 mm) were eliminated [26]. In this image, blood vessels with magnification-corrected calibers smaller than 60 μm were also eliminated. Supplemental Figure 1E The 6 biggest arterioles and venules are shown, from which central artery and vein equivalents were calculated [21]. [file 12886_2022_2428_MOESM2_ESM.docx]
